# Third follow-up of the Cardiovascular Risk Factors, Aging and Dementia (CAIDE) cohort investigating determinants of cognitive, physical, and psychosocial wellbeing among the oldest old: the CAIDE85+ study protocol

**DOI:** 10.1186/s12877-020-01617-5

**Published:** 2020-07-10

**Authors:** Mariagnese Barbera, Jenni Kulmala, Inna Lisko, Eija Pietilä, Anna Rosenberg, Ilona Hallikainen, Merja Hallikainen, Tiina Laatikainen, Jenni Lehtisalo, Elisa Neuvonen, Minna Rusanen, Hilkka Soininen, Jaakko Tuomilehto, Tiia Ngandu, Alina Solomon, Miia Kivipelto

**Affiliations:** 1 Institute of Clinical Medicine, Department of Neurology, University of Eastern Finland, P.O. Box 1627, 70211 Kuopio, Finland; 2 Public Health Promotion Unit, Finnish Institute for Health and Welfare, P.O. Box 30, 00271 Helsinki, Finland; 3 School of Health Care and Social Work, Seinäjoki University of Applied Sciences, Seinäjoki, Finland; 4 Division of Clinical Geriatrics, Center for Alzheimer Research, Care Sciences and Society (NVS), Karolinska Institutet, Karolinska Universitetssjukhuset, Karolinska Vägen 37 A, QA32, Stockholm, Sweden; 5 Institute of Public Health and Clinical Nutrition, University of Eastern Finland, P.O. Box 1627, 70211 Kuopio, Finland; 6 Joint Municipal Authority for North Karelia Social and Health Services (Siun Sote), Central Hospital, Tikkamäentie 16, 80210 Joensuu, Finland; 7 Neurocenter Finland, Department of Neurology, Kuopio University Hospital, Puijonlaaksontie 2, 70210 Kuopio, Finland; 8 Department of Public Health, University of Helsinki, PO BOX 20, 00014 Helsinki, Finland; 9 Diabetes Research Group, King Abdulaziz University, Jeddah, 21589 Saudi Arabia; 10 Ageing Epidemiology Research Unit, School of Public Health, Imperial College London, Charing Cross Hospital, St Dunstan’s Road, London, W6 8RP UK

**Keywords:** Ageing, CAIDE, Cognitive decline, Dementia, Disability, Longevity, Longitudinal cohort study, Midlife risk factors, Physical functioning, Protocol

## Abstract

**Background:**

The oldest old is the fastest growing age group worldwide and the most prone to severe disability, especially in relation to loss of cognitive function. Improving our understanding of the predictors of cognitive, physical and psychosocial wellbeing among the oldest old can result in substantial benefits for the individuals and for the society as a whole.

The Cardiovascular Risk Factors, Aging and Dementia (CAIDE) study investigated risk factors and determinants of cognitive impairment in a population-based longitudinal cohort, which was first examined between 1972 and 1992, when individuals were in their midlife, and re-assessed in 1998 and 2005–2009. Most of the study participants are currently aged 85 years or older. We aim to re-examine the cohort’s survivors and gain further insights on the mechanisms underlying both cognitive and overall healthy ageing at old age.

**Methods:**

CAIDE85+ is the third follow-up of the CAIDE study participants. All individuals still alive and living in the Kuopio and Joensuu areas of Eastern Finland, from the original CAIDE cohort (two random samples, *N* = 2000 + ~ 900), will be invited to a re-examination. The assessment includes self-reported data related to basic demographics and lifestyle, as well as psychosocial and physical health status. Cognitive and physical evaluations are also conducted. Blood biomarkers relevant for dementia and ageing are assessed.

Primary outcomes are the measurements related to cognition and daily life functioning (CERAD, Trail Making Test-A, Letter-Digit Substitution Test, Clinical Dementia Rating and Activities of Daily Living). Secondary endpoints of the study are outcomes related to physical health status, psychosocial wellbeing, as well as age-related health indicators.

**Discussion:**

Through a follow-up of more than 40 years, CAIDE85+ will provide invaluable information on the risk and protective factors that contribute to cognitive and physical health, as well as ageing and longevity.

**Study registration:**

The present study protocol has been registered at https://clinicaltrials.gov/ (registration nr NCT03938727, date 03.05.2019).

## Background

The rapid growth of the oldest section of the population is setting new challenges for modern societies worldwide. The oldest old, often defined as people aged 85 years or more [1], is currently the fastest-growing age group in the developed countries, as well as the most prone to disabling conditions and use of long-term care services [2]. Dementia, the main reason for institutionalisation among the oldest old [3], and Alzheimer’s disease (AD), its most common cause, are currently one of the world’s key global public health priorities, as well as a major social and economic burden [4]. Approximately 25–30% of people in their early 90s, 50% of those in their late 90s, and 60% of those aged 100 years or more live with AD or other forms of dementia [5]. Reducing the risk of developing dementia and improving the overall health status, psychosocial wellbeing, and the quality of life of the oldest old would have important individual and public health, as well as societal and economic benefits.

Several midlife modifiable cardiovascular risk factors, including hypertension, hypercholesterolemia, diabetes mellitus, obesity, smoking, physical inactivity, and unhealthy diet, have been linked to dementia at older ages [4, 6, 7], creating opportunities for prevention [8, 9]. Furthermore, a strong association of poor physical health status with dementia has been found among the oldest old [10]. Potential predictors of longevity, such as healthy lifestyle at midlife [11], higher socioeconomic status [12], and psychosocial support [13] have also been proposed. However, prospective studies with repeated assessments extending to the oldest old are still relatively rare.

### Rationale

The Cardiovascular Risk Factors, Aging and Dementia (CAIDE) study is a population-based cohort study initiated in 1998 to investigate the potential role of midlife modifiable risk and protective factors in the development of dementia [14, 15]. The re-examinations carried out so far have provided essential knowledge on the role of vascular and lifestyle risk factors [15–20], including interactions with genetics [14]. Using the study data, the CAIDE Dementia Risk Score was the first tool developed to predict the risk of late-life dementia based on lifestyle and cardiovascular risk factors at midlife. It is currently used as a research tool [21–23], and it enabled the selection of individuals at increased risk of cognitive decline in the first successful larger-scale and longer-term multidomain lifestyle trial in the dementia prevention field [24].

### Aims

Ten years after the second follow-up, most of the CAIDE participants belong now to the oldest old age group. The new CAIDE85+ study, which is the third follow-up within this cohort, aims to further investigate risk and protective factors for dementia, as well as improve our understanding of the life-course factors affecting the ageing process. To this aim, the cognitive and physical health status, daily life functioning, as well as lifestyle, psychosocial wellbeing, and quality of life, are investigated in this cohort.

## Methods

### Overall design of the CAIDE study

CAIDE is a prospective longitudinal cohort study started in 1998 in Eastern Finland; CAIDE85+ is its third re-examination. CAIDE and CAIDE85+ have been approved by the Ethics Committee, Hospital District of Northern Savo (Finland), and the CAIDE85+ study protocol has been registered at https://clinicaltrials.gov/ (registration nr NCT03938727, date 03.05.2019).

**Fig. 1:**
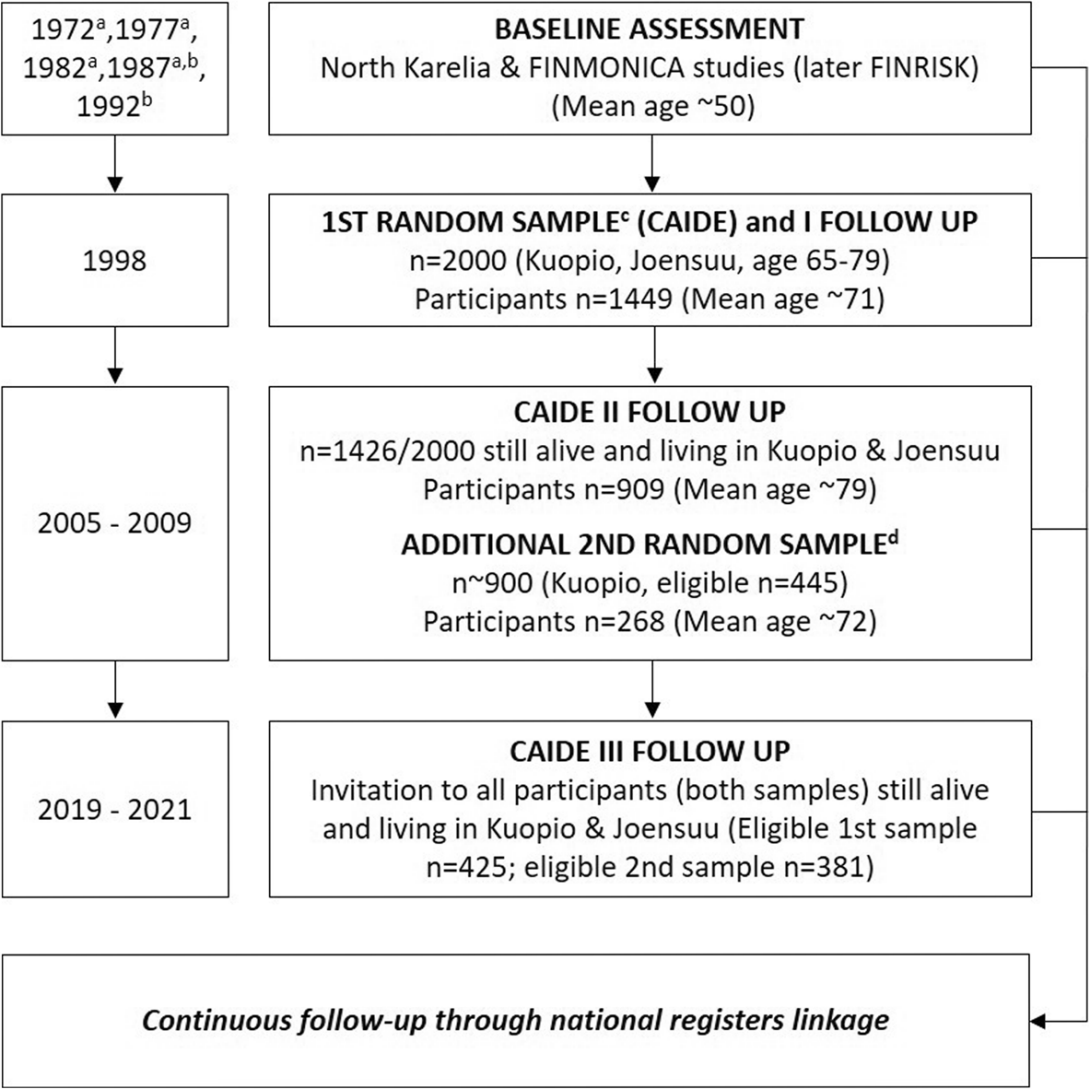
CAIDE study diagram. a: participants from this cohort were included in the first random sample identified in 1998; b: participants from this cohort were included in the second random sample identified in 2006; c: includes participants from the 1972, 1977, 1982, and 1987 midlife cohorts; d: includes participants from the 1987 and 1992 midlife cohorts

In 1998, a first random sample of potential participants was identified from Finnish population-based cross-sectional surveys (North Karelia project in 1972 and 1977, FINMONICA study in 1982 and 1987, Fig. 1) [25–28]. A total of 2000 individuals living in the Kuopio and Joensuu areas of Eastern Finland and assessed at midlife (average age = 50.4 ± 6 years) were invited to the first re-examination of the CAIDE study (Fig. 1), and 1449 agreed to participate. The second re-examination took place between 2005 and 2008. A total of 1426 of the original 2000 participants were still alive and living in the Kuopio and Joensuu areas, and a total of 909 agreed to participate (Fig. 1).

In 2006, a second random sample of 897 people was identified from the 1987 FINMONICA and 1992 FINRISK study cohorts. A total of 445 individuals were eligible (still alive, living in Kuopio and not already invited to CAIDE), and 268 were re-examined between 2006 and 2009 (Fig. 1).

The CAIDE85+ study presented here includes both CAIDE random samples; recruitment and re-examinations started in June 2019 and will continue until 2021, more than 40 years after the baseline (midlife) assessments.

### Population, recruitment, and inclusion/exclusion criteria

Every individual who was part of a CAIDE random sample and is still alive and living in the Kuopio or Joensuu areas at the time of recruitment is considered eligible for participation. No other inclusion or exclusion criteria are applied.

Eligible participants are identified through the national population registry and invited by post. From the first CAIDE sample, 425 eligible individuals were identified in May 2019; from the second CAIDE sample, 381 eligible individuals were identified in September 2019. Based on previous recruitment rate, and considering the frailty level as well as the high rate of comorbidities in this age group, 500 participants are estimated to be enrolled in the present follow-up.

Participants are asked to nominate a study informant, i.e. a relative or a friend who is at least 18 years old and who may or may not live with the participant, to provide information related to physical and cognitive health of the participant. The recruitment of a study informant is encouraged and supported but is not a binding requirement to be included in the study.

### Data collection

The data collection is conducted through questionnaires, interview, and physical and cognitive examinations by a research nurse trained for the study procedures. Based on the participant’s preferences, the visit may take place either at the research site or at the participant’s domicile.

At the baseline (midlife) visit, data were collected regarding socio-demographics (age, sex, education, marital status, occupation, income), vascular risk factors (e.g. blood pressure, blood lipids, body mass index), lifestyle (e.g. smoking, alcohol, physical activity, diet), psychosocial factors, and physical health status. At the CAIDE re-examinations, a detailed cognitive evaluation was conducted, including a 3-step protocol for the diagnosis of dementia [29], and apolipoprotein E genotype was assessed. In the present CAIDE85+ re-examination, data collection has been harmonised with previous follow-ups, with some adaptations and additions specifically relevant for the 85+ population (e.g. addition of the frailty index and indicators of malnutrition), or recent scientific developments related to the aims of the study (e.g. additional assessment of the quality of sleep and oral health). In Table 1, a full summary and comparison of the assessments carried out at each examination is presented.

**Table 1:** Summary of the assessments in the CAIDE study

Data collected and test/scale	Midlife-baseline examination (1972–1992)	1st late-life re-examination (1998)	2nd late-life re-examination (2005–2009)	3rd re-examination CAIDE85+ (2019–2021)
Socio-demographics^a^ (e.g. age; sex; self-reported education, marital status, occupation and income)	**X**	**X**	**X**	**X**
Lifestyle^a^ (questions about e.g. diet, physical exercise, smoking, leisure activities)	**X**	**X**	**X**	**X**
Anthropometric measurements (weight, height, BMI, waist circumference, blood pressure)	**X**	**X**	**X**	**X**
Blood markers^a^ (e.g. lipids, glucose, CRP, creatinine)	**X**	**X**	**X**	**X**
Medical history (self-reported)	**X**	**X**	**X**	**X**
Medical history (national registers)	**X**	**X**	**X**	**X**
***Cognition***				
CERAD [30]	**–**	**–**	**X**	**X**
MMSE [31]	**–**	**X**	**Part of CERAD**	**Part of CERAD**
Immediate word recall (3 word-lists)^b^ [32]	**–**	**X**	**1 list**	**–**
Category fluency test [33]	**–**	**X**	**Part of CERAD**	**Part of CERAD**
Purdue Peg board [34]	**–**	**X**	**X**	**–**
Trail Making Test - part A [35]	**–**	**–**	**X**	**X**
Stroop [36]	**–**	**X**	**X**	**–**
Letter Digit Substitution Test [37]	**–**	**X**	**X**	**X**
Diagnosis of dementia or MCI (3-step protocol [29])	**–**	**X**	**X**	**–**
Diagnosis of dementia or MCI (national registers)	**–**	**X**	**X**	**X**
Daily life functioning (questions)	**X**	**X**	**X**	**X**
Daily life functioning (ADL: Katz Index and Lawton and Brody Scale [38–40])	**–**	**–**	**–**	**X**
Daily life functioning (CDR [41, 42])	**–**	**–**	**X**	**X**
Physical Functioning (SPPB [43])	**–**	**–**	**–**	**X**
Stress^a^ (questions)	**X**	**X**	**X**	**X**
Anxiety (6-item STAI [44])	**–**	**X**	**X**	**X**
Personality-related factors (anger expression [45], cynical distrust [46], sense of coherence [47], ways of coping [48])	**–**	**X**	**–**	**–**
Social Network (questions)	**–**	**X**	**X**	**Shortened**
Subjective memory^a^ (questions)	**–**	**X**	**X**	**X**
Hopelessness (questions)	**X**	**X**	**X**	**X**
Depression (BDI [49])	**–**	**X**	**X**	**X**
Significant life events (questions)	**–**	**X**	**X**	**Shortened**
Health Related Quality of Life (RAND 36 [50])	**–**	**–**	**–**	**X**
Frailty (Fried phenotype [51])	**–**	**–**	**–**	**X**
Malnutrition (MNA short version [52])	**–**	**–**	**–**	**X**
Sleep quality (Pittsburgh Sleep Index [53])	**–**	**–**	**–**	**X**
Oral health (questions)	**–**	**–**	**–**	**X**
Medication use (self-reported and national registers)	**–**	**X**	**X**	**X**
APOEƐ4 genotyping	**–**	**X**	**X**	**–**

*ADL* Activity of daily living, *APOEƐ4* Apolipoprotein E Ɛ4 allele, *BDI* Beck depression inventory, *BMI* body mass index, *CDR* clinical dementia rating, *CERAD* Consortium to Establish a Registry for Alzheimer’s Disease test battery, *CRP* C-reactive protein, *MCI* Mild cognitive impairment, *MMSE* Mini-Mental State Examination, *MNA* mini nutritional assessment, *SPPB* Short physical performance battery, *RAND36* RAND 36-Item Health Survey 1.0, *STAI* State trait anxiety inventory

^a^ some differences in assessments may exist among different re-examinations

^b^ of the 3 word-lists used in 1998, only one was repeated in 2005–2008

### Primary outcomes

The primary outcomes of CAIDE85+ are cognitive performance, daily life functioning, dementia and mild cognitive impairment (MCI).

*Cognitive performance* is evaluated using the Finnish version of the Consortium to Establish a Registry for Alzheimer’s Disease (CERAD) test battery, which was carried out also during the 2005–2008 re-examination [30]. This includes: *Modified 15-item Boston Naming Test* [54], *Category fluency* [33], *Mini-Mental State Examination* [31], *10-Word Recall Task (10 word learning, recall and recognition)* [30], *Constructional Praxis* [55] and *Recall* [56], and *Clock Drawing Test* [57]. In addition to CERAD, the *Trail Making Test part A* [35] and the *Letter-Digit Substitution Test* [37] are also used.

*Daily life functioning. Functioning level* is measured through the *Clinical Dementia Rating (CDR)* [41, 42], for which a semi-structured interview with the participant and the study informant will be conducted. *Activities of Daily Living (ADL)* is assessed using both the Katz Index of Independence in Activities of Daily Living and the Lawton-Brody Scale of Instrumental Activities of Daily Living [38–40].

*Dementia diagnoses* (including the type of dementia) is ascertained from medical records and data linkage to national registers, such as hospital discharge, outpatient, drug reimbursement, and causes of death registers. *MCI* is defined based on standard criteria [58] which include: abnormal cognitive test performance; subjective memory complaint; and minimal or no impairment in activities of daily living.

### Secondary outcomes

The study’s secondary outcomes include multimorbidity, frailty, mobility and functional performance, physical activity, psychosocial wellbeing, oral health, nutrition, sleep quality and health-related quality of life.

*Multimorbidity* is defined based on medical history data from e.g. questionnaires, physical assessments, medical records and national registers.

*Frailty* is defined with the Fried Frailty phenotype [51], which includes: unintentional weight loss; self-reported exhaustion; weakness by grip strength; slow walking speed; and low physical activity. *Mobility and functional performance* is assessed based on self-reported data on mobility and fitness, as well as an objective and physical assessment, which includes the Short Physical Performance Battery [43] and the maximal isometric handgrip strength measured by a hand-held adjustable dynamometer.

*Physical activity* is assessed based on self-reported questionnaires about frequency and type of activities carried out.

Assessment of *Psychosocial wellbeing* cover several domains. *Hopelessness*, *Social network and interactions*, and *Subjective memory* [59] is investigated through questionnaires. *Anxiety* is assessed with the self-reported validated State Trait Anxiety Inventory [44]. *Depressive symptoms* are self-reported through the Beck Depression Inventory [49]. *Significant life events* is recorded using a brief questionnaire based on the Swedish National study on Aging and Care [60] questionnaire, including major life events with potential impact on physical, psychological, and emotional status [61].

*Oral health* is appraised through a self-report questionnaire inquiring about oral health status and use of oral healthcare services. *Nutritional status* is investigated using a questionnaire on food consumption frequency, including alcohol consumption and drinking patterns, as well as the Mini Nutritional Assessment (short-form) [52], a validated tool commonly used to screen for and estimate risk of malnutrition in older adults.

*Sleep quality* is assessed using the Pittsburgh Sleep Quality Index [53].

*Health-related quality of life* is measured with the RAND 36-Item Health Survey 1.0. [50]

### Other data collected

*Sociodemographic factors* include age, sex, self-reported data on marital status, living/domicile setting, yearly income, and work history. *Other health-related data* cover current medication and medical history; self-reported use of healthcare services; self-reported type and frequency of leisure activities; self-reported use of the computer and the Internet; biometrics such as blood pressure, body mass index, and waist-hip ratio; smoking habits; information from medical records, such as diagnoses, hospitalisations, and other relevant health events. Dates and causes of death will be obtained from national registers.

Fasting blood samples are collected for assessment of plasma glucose, glycated haemoglobin (HbA1c); serum total-, high-density lipoprotein-, and low-density lipoprotein cholesterol, and triglycerides; c-reactive protein; and creatinine. Blood samples will also be stored for future measurement of other dementia- and aging-related biomarkers*.*

### Ethical considerations

The CAIDE85+ study was approved by the Ethics Committee, Hospital District of Northern Savo (Finland). Prior to the study enrolment, written informed consent is obtained from each participant, as well as their informant, if available. If a participant has received a diagnosis of AD or other dementia prior to the time of enrolment, the ability to autonomously consent to the study is assessed by the research nurse. If the participant is deemed unable to provide and informed consent, a legally acceptable representative (LAR), is identified with the help of the participant, to co-sign the consent form. Failing to identify a LAR for a participant unable to provide an informed consent precludes the participation in the study.

### Data analysis

Cross-sectional analyses and descriptive statistics, including potential comparisons with other 85+ cohorts available in the literature will be carried out. Longitudinal analyses combining repeated measures data from the first two follow-ups, as well as baseline (midlife), will also be conducted, where applicable. Appropriate statistical methods will be used to analyse the data, including proportional hazards modelling and appropriate regression models (e.g. Kaplan-Meier method, logistic, linear, ordinal, Cox and Poisson regression models). To analyse different trajectories of predictor variables across multiple waves of data, path analyses and structural equation modelling will be used. Mortality will be taken into account in longitudinal analyses. Novel statistical methods (e.g. machine learning) [62] may also be tested for multifactorial prediction. Statistical significance will be defined at *p* < 0.05 and relevant guidelines (e.g. STROBE [63], TRIPOD [64]) will be used for results interpretation and reporting.

## Discussion

In the last century, life expectancy has persistently increased to levels earlier often considered unattainable. Despite recent findings reporting declining life expectancy in the United States and United Kingdom [65, 66], globally the projections for the next decades confirm this strong trend [67]. As a consequence, the oldest olds are now by far the fastest growing age group, in developed countries in particular [68]. Such shift in the age distribution of the population will affect not only healthcare systems, but also, more in general, the way in which society will cope with ageing-related matters. Living longer does not necessarily mean living better, as life expectancy and quality of life do not always go hand in hand [2]. However, evidence suggests that ageing processes may be modifiable, and people could live longer without increased disabilities [1].

Dementia, AD, and cognitive impairment constitute, nonetheless, a major ageing-related social, economic, and public health concern. With the ageing of populations, the number of people living with dementia is also expected to increase rapidly [69]. Additional efforts and resources are, therefore, needed to improve our understanding healthy ageing and its determinants.

The oldest old include individuals often vulnerable to and impaired by severe disabilities. Thus, extensive investigation of this age group poses specific ethical and logistic challenges [70], which can have a great impact on recruitment success, study implementation, and data interpretation. Altogether, studies on the oldest old are scarce and often conducted on relatively small population samples. In the last few decades, some large cohort studies have been conducted focusing on the broad determinants of health in the oldest old [71–75]. However, in none of them the participants have been followed-up starting from midlife. Furthermore, recruitment in most of these studies was completed from the beginning of the 1990s to the early 2000s. In a society in constant cultural evolution and marked by substantial lifestyle changes linked to increased access to new technologies, the current nonagenarians are likely to be a rather different population group than only a few decades ago.

The CAIDE85+ study represents a rare opportunity to delve into the determinants of cognitive and physical health within a current population of oldest old, who have been followed for decades, since their midlife until old age. Such a long follow-up time is rarely achievable in observational studies. A wide range of outcomes from more specific cognitive measures to medical, physical and psychological parameters is investigated, using, whenever possible, methods specifically designed and particularly relevant for this age group.

Through a thorough investigation of the cognitive and physical functioning, as well as the incidence of dementia and cognitive impairment, this study will contribute to improve the knowledge on risk and protective factors for cognitive impairment and underlying mechanisms, which may help reduce the detrimental impact of dementia on both individuals and the society. Findings from this study will also enable us to better understand the role of lifestyle and medical factors that contribute to good physical status, successful cognitive ageing, as well as increased quality of life and healthy ageing in general at the oldest old ages. It is expected that different risk factors would play a key role in different age groups. This has practical implications in planning preventive intervention programmes, developing new therapeutic strategies, and educating the general population, as well as healthcare professionals. Therefore, findings from the study may also help develop interventions focused on promotion of healthy ageing, and plan individually tailored healthcare for the oldest old age groups.

The high mortality and morbidity rates, as well as the high prevalence of cognitive impairment expected in a population of oldest old are potential limitations in this study, but several provisions have been put in place to reduce their impact. First, the use of thorough and up-to-date Finnish health registers will allow us to collect essential data that could not be obtained otherwise. Second, in order to meet the needs of all participants, the study visits have been designed to have a high degree of flexibility both in terms of location (research site or participant’s domicile) and timing (over one or more appointments). In particular, the possibility of conducting home visits and, if needed, in more than one session, helps reduce the burden and ease the participation also for individuals who are more disabled. Finally, assessing cognition using well-validated and widely used tests help compensate for potential inaccuracies in the collection of self-reported data.

In conclusion, by providing more detailed insight on the medical, lifestyle, psychosocial and physical-related factors contributing to cognitive health; quality of life; and psychosocial wellbeing among the oldest old, our findings will produce urgently needed information for healthcare workers and policy makers to better promote health and independent living among this rapidly growing age group.

## Data Availability

Data will not be made publicly available due to Ethical requirements. External collaborators can apply to the CAIDE study steering group for pseudonymised datasets and/or samples. Application must be submitted to Assoc. Prof. Alina Solomon (alina.solomon@uef.fi).

## Abbreviations

AD: Alzheimer’s Disease
ADL: Activities of daily living
CAIDE: Cardiovascular Risk Factors, Aging and Dementia
CDR: Clinical Dementia Rating
CERAD: Consortium to Establish a Registry for Alzheimer’s Disease
FINMONICA: Finnish Multinational MONItoring of trends and determinants in CArdiovascular disease study
FINRISK: National FINRISK Study
LAR: Legally acceptable representative
MCI: Mild cognitive impairment
STROBE: Statement for Strengthening the Reporting of Observational Studies in Epidemiology
TRIPOD: Statement for Transparent Reporting of a multivariable prediction model for Individual Prognosis Or Diagnosis
